# Four transcription profile–based models identify novel prognostic signatures in oesophageal cancer

**DOI:** 10.1111/jcmm.14779

**Published:** 2019-11-19

**Authors:** Tongyan Liu, Panqi Fang, Chencheng Han, Zhifei Ma, Weizhang Xu, Wenjia Xia, Jingwen Hu, Youtao Xu, Lin Xu, Rong Yin, Siwei Wang, Qin Zhang

**Affiliations:** ^1^ Department of Thoracic Surgery The Affiliated Cancer Hospital of Nanjing Medical University & Jiangsu Cancer Hospital & Jiangsu Institute of Cancer Research, Jiangsu Key Laboratory of Molecular and Translational Cancer Research Nanjing China; ^2^ Department of Scientific Research The Affiliated Cancer Hospital of Nanjing Medical University & Jiangsu Cancer Hospital & Jiangsu Institute of Cancer Research, Jiangsu Key Laboratory of Molecular and Translational Cancer Research Nanjing China; ^3^ Department of Clinical Pharmacy School of Basic Medical Sciences and Clinical Pharmacy China Pharmaceutical University Nanjing China; ^4^ The Fourth Clinical College of Nanjing Medical University Nanjing China; ^5^ Jiangsu Biobank of Clinical Resources Nanjing China

**Keywords:** machine learning, oesophageal cancer, prognostic signature, transcription profile

## Abstract

Oesophageal cancer (ESCA) is a clinically challenging disease with poor prognosis and health‐related quality of life. Here, we investigated the transcriptome of ESCA to identify high risk‐related signatures. A total of 159 ESCA patients of The Cancer Genome Atlas (TCGA) were sorted by three phases. In the discovery phase, differentially expressed transcripts were filtered; in the training phase, two adjusted Cox regressions and two machine leaning models were used to construct and estimate signatures; and in the validation phase, prognostic signatures were validated in the testing dataset and the independent external cohort. We constructed two signatures from three types of RNA markers by Akaike information criterion (AIC) and least absolute shrinkage and selection operator (LASSO) Cox regressions, respectively, and all candidate markers were further estimated by Random Forest (RFS) and Support Vector Machine (SVM) algorithms. Both signatures had good predictive performances in the independent external oesophageal squamous cell carcinoma (ESCC) cohort and performed better than common clinicopathological indicators in the TCGA dataset. Machine learning algorithms predicted prognosis with high specificities and measured the importance of markers to verify the risk weightings. Furthermore, the cell function and immunohistochemical (IHC) staining assays identified that the common risky marker FABP3 is a novel oncogene in ESCA.

## 1 INTRODUCTION

Oesophageal cancer (ESCA) is the eighth leading cancer and the sixth highest cause of cancer death worldwide.^1^ In total, 17 290 newly diagnosed cases and 15 850 oesophageal cancer deaths were estimated in 2018.^2^ ESCA has two main histological types, squamous cell carcinoma (SCC) and adenocarcinoma, in which SCC accounts for the most of ESCA cases of China.^3^ The poor prognosis of ESCA is partially due to lack of effective early diagnosis and post‐surgeon surveillance.^4^ At present, the tumour‐node‐metastasis (TNM) staging system is the only well‐recognized stratification system for treatment decisions.^5^ However, TNM staging fails to assess the clinical outcome in a great number of patients.^6^ Patients with the same stage category receive similar treatments, but their clinical outcome varies greatly.^7^ Therefore, there is a pressing need to identify reliable prognostic factors for prediction in ESCA patients.

Second‐generation sequencing characterizing ESCA transcriptomes has revealed mounts of molecular markers.^8^, ^9^ Several studies have shown that messenger RNAs (mRNAs), microRNAs (miRNAs) and long non‐coding RNAs (lncRNAs) could become predictive signatures of survival and treatment results with good performance.^8^, ^10^, ^11^ Li et al have found a three‐lncRNA signature is associated with ESCC patients’ survival status.^8^ Xiong et al have revealed a multi‐RNA–based classifier to improve prognosis prediction of colorectal cancer.^12^ Furthermore, emerging evidence has shown that unique miRNA panel could assist in early diagnosis of virus‐related hepatocellular carcinoma,^10^, ^13^ prognosis for patients underwent esophagectomy,^14^ and prediction for trastuzumab benefit in patients with HER2‐positive metastatic breast cancer.^11^ However, whether the signature combining different types of RNA markers could have better performance in the ESCA prognostic prediction remains unknown.

To date, Cox and penalized Cox regressions were widely used for the stable feature selection.^15^, ^16^ Smyth et al identified a seven‐gene signature to improve prognostic risk stratification in chemotherapy treated gastroesophageal cancer patients by using standard Cox regression model.^17^ In addition, machine learning, a branch of artificial intelligence, was successfully applied in screening biomarkers correlated with cancer diagnosis, prognosis and treatment.^18^ In another study, selecting with least absolute shrinkage and selection operator (LASSO) Cox and Support Vector Machine (SVM) algorithms, Qiu et al 19 constructed a three‐CpG signature in predicting recurrence for patients with early‐stage hepatocellular carcinoma. Thus, combining semi‐parametric and machine learning algorithms has the potential to enhance the accuracy of the present prognostic indicators.

Here, we constructed a multi‐marker signature based on exploring mRNA, lncRNA and miRNA profiles of ESCA with 2 regularization semi‐parametric algorithms. Machine learning algorithms were then used to estimate the importance of included markers, which validated the risk weightings of Cox regressions. Additionally, loss‐of‐function and immunohistochemical (IHC) staining assays identified the oncogenic function of the novel ESCA marker FABP3.

## 2 MATERIAL AND METHODS

### 2.1 Data collection and processing

We obtained 159 ESCA patients’ clinical and sequencing data, including the RNA‐sequencing and miRNA‐sequencing datasets, from the TCGA (The Cancer Genome Atlas) data portal (April 2016; https://portal.gdc.cancer.gov/). Based on the annotation information of the Ensemble GRCh37 genome, we identified 10 617 long non‐coding RNAs and 18 687 protein coding genes. Differentially expressed lncRNAs, mRNAs and miRNA between ESCA and adjacent normal tissues were screened by edgeR package in R software (version 3.5.1), and |log2 FoldChange| ≥ 2 and FDR < 0.01 were considered significant. After normalization within edgeR, the expression profiles could be used for the next processing.

### 2.2 Study design

In this study, we included three phases to identify and validate risk‐related transcripts for patients with ESCA. The TCGA ESCA cohort was randomly divided into the training (79 samples) and internal testing (80 samples) datasets. The random assignment was conducted blindly by a computerized random assignment sequence. The association between differentially expressed genes (DEGs) and overall survival (OS) was assessed with the Kaplan‐Meier survival analysis. In the training phase, Akaike information criterion (AIC) adjusted Cox regression, least absolute shrinkage and selection operator (LASSO) adjusted Cox regression, Random Forest (RFS) and Support Vector Machine (SVM) algorithms were used to identify the potential prognostic markers.

Coefficients from adjusted Cox regressions were used to construct prognostic signatures. Random Forest‐Feature Selection (RFS‐FS) and Support Vector Machine‐Recursive Feature Elimination (SVM‐RFE) were applied to rank marker importance.^20^, ^21^ Additionally, the risk difference between high‐risk and low‐risk patients was estimated with Kaplan‐Meier survival analyses. The area under the curve (AUC) of the receiver operating characteristic (ROC) curve was calculated to estimate the performance of each model.

### 2.3 Cell culture, RNA extraction and qRT‐PCR analysis

Two oesophageal squamous cell carcinoma (ESCC) cell lines KYSE150 and Eca109 were obtained from the American Type Culture Collection (ATCC). Cells were cultured in RMPI1640 medium (KeyGene), supplemented with 10% FBS with 100 U/mL penicillin and 100 mg/mL streptomycin. All cell lines were grown in humidified air at 37°C with 5% CO_2_. Cell cultures were occasionally tested for mycoplasma (last tested in June 2018). The cells used in experiments were within 10 passages from thawing. RNA extraction and qRT‐PCR were performed as described previously.^22^ We used β‐actin and U6 as internal controls and tested marker expression levels by qRT‐PCR. We used the following primer sequences, β‐actin: *CGCTCTCTGCTCCTCCTGTTC* (Forward) and *ATCCGTTGACTCCGACCTTCA*C (Reverse); U6: *CTCGCTTCGGCAGCACA* (Forward) and *AACGCTTCACGAATTTGCGT* (Reverse); FABP3: *GGCACCTGGAAGCTAGTGG* (Forward) and *CTGCCTGGTAGCAAAACCC* (Reverse); AC010776.2: *AACCAACCCTCAAAGATTCGC* (Forward) and *AACCAACCCTCAAAGATTCGC* (Reverse); AC119424.1: *GGGCCAATCACGAAGGAGAA* (Forward) and *CTTCCTGTGGTGATGCCGAT* (Reverse); GK‐IT1: CTCCAACTGAGCAGCACACA (Forward) and *ATTCCTTGAGCCCAGTGACAG* (Reverse); BHLHA15: *GCGGACAAGAAGCTCTCCAAGA* (Forward) and *TGGTAGTGCTGGTAGAGCTTGG* (Reverse); CLCNKB: *CCCTCTACAAGACCAGTTTCCG* (Forward) and *GCTGACAGAAGAGGTAAGCGCT* (Reverse). The miRNA primers of miR‐4664 and miR‐615 were provided by RiboBio.

### 2.4 Patients and tissue samples

We gained access to primary oesophageal cancer tissues through JiangSu Cancer Hospital Biobank. All tumours were confirmed by experienced pathologists. Written informed consent was obtained from all patients. Collection of human tissue samples was conducted in accordance with the International Ethical Guidelines for Biomedical Research Involving Human Subjects. This study was approved by the Ethics Committee of the JiangSu Cancer Hospital and was performed in accordance with the provisions of the Ethics Committee of Nanjing Medical University. This study was approved by the Nanjing Medical University.

### 2.5 Cell proliferation, migration and apoptosis assays

Cell proliferation was examined using EdU assay (RiboBio), and Real Time xCELLigence Analysis (RTCA) system following the research protocol afforded by the manufacturer (Roche Applied Science and ACEA Biosciences). Cell migration ability was conducted using RTCA and 24‐well transwells (8 μm pore size, Millipore). Cell invasion assays were examined using 24‐well transwells coated with 1ml/mL Matrigel (8 μm pore size, BD Science). Apoptosis assays were conducted with FACSCanto II and Annexin V R‐PE 20 tests (BD Science).

### 2.6 Small interference RNA construction and cell transfection

The small interference RNAs (siRNAs) were provided by RealGene Technologies. Scramble control or FABP3 siRNAs were transfected into lung adenocarcinoma cells using RNAiMAX (Invitrogen) according to the manufacturer's instructions. We used the following siRNA sequences: SiFABP3‐1 forward sequence *CUACCACAAUCAUCGAAAATT*; SiFABP3‐1 reverse sequence *UUUUCGAUGAUUGUGGUAGGC*; SiFABP3‐2 forward sequence *GCAAGAAUUUCGAUGACUATT*; SiFABP3‐2 reverse sequence *UAGUCAUCGAAAUUCUUGCTG*.

### 2.7 Tissue microarrays

Tissue microarray (TMA) was constructed as described previously.^22^ ESCC tumour and normal tissues of 39 cases were used to construct the TMA. All included tissue samples were confirmed by experienced pathologists and conducted in accordance with the International Ethical Guidelines for Biomedical Research Involving Human Subjects. This study was approved by the Ethics Committee of the Nanjing Medical University Affiliated Cancer Hospital and was performed in accordance with the provisions of the Ethics Committee of Nanjing Medical University. Immunohistochemical (IHC) staining was performed to detect FABP3 expression in TMA using FABP3 antibody (Catalog number: 60280‐1‐Ig, Proteintech Group), and IHC scores were estimated by two pathologists, respectively (Table S1).

### 2.8 Statistical analysis

GraphPad prism 8 and R software version 3.5.1 were used to plot the figures. The packages of glmnet, randomforest and e1071 in R were used to perform the LASSO Cox regression, Random Forest and SVM models. The performance of the nomogram for the test dataset by the concordance index was measured using the rms and Hmisc R packages. For survival analysis, overall survival was calculated using the Kaplan‐Meier method and the log‐rank test by the survival R package. Additionally, the time‐dependent receiver operating characteristic (ROC) curves were plotted by the timeROC R package, and the bootstrap method was used to compare the signature for area under the ROC (AUC) curve. All *P* values were two sided, and *P* value < .05 was considered to be statistically significant.

## 3 RESULTS

### 3.1 Clinicopathological features of patients

The baseline clinicopathological features of ESCA training and testing datasets from TCGA were shown in Table 1. A total of 159 ESCA patients, including 79 ESAD (oesophageal adenocarcinoma) and 80 ESCC samples, were randomly classified into training set (n = 79, mean age: 62.6 ± 12.6 years) and internal testing set (n = 80, mean age: 63.3 ± 11.4 years), respectively. Furthermore, all included patient specimens were underwent both RNA and miRNA sequencing. As we expected, the clinicopathological characteristics in training and testing datasets were well balanced in age (*P* = .806), gender (*P* = .974), local invasion (*P* = .654), lymph node metastasis (*P* = .498), distant metastasis (*P* = .165) and pathological type (*P* = .303).

**Table 1:** Clinical features for the ESCA patients in the training and testing datasets

Characteristics	Training dataset n = 79 (50%)	Testing dataset n = 80 (50%)	*P* value
Age (years)			
＜60	35	38	.806
≥60	44	42	
Gender			
Male	67	69	.974
Female	12	11	
Local invasion			
T1	16	10	.654
T2	20	20	
T3	41	45	
T4	2	3	
TX		2	
Lymph node metastasis			
N0	37	29	.498
N1	31	40	
N2	5	5	
N3	4	2	
NX	2	4	
Distant metastasis			
M0	65	61	.165
M1	4	11	
MX	10	8	
Type			
ESAD	43	36	.303
ESCC	36	44	

Note

*P* value from chi‐squared test or Fisher's exact test for nominal categories.

Abbreviations: ESAD, oesophageal adenocarcinoma; ESCC, oesophageal squamous cell carcinoma; Mx, uncertain M stage; Nx, uncertain N stage; Tx, uncertain T stage.

### 3.2 Selection of candidate prognostic markers

**Figure 1:**
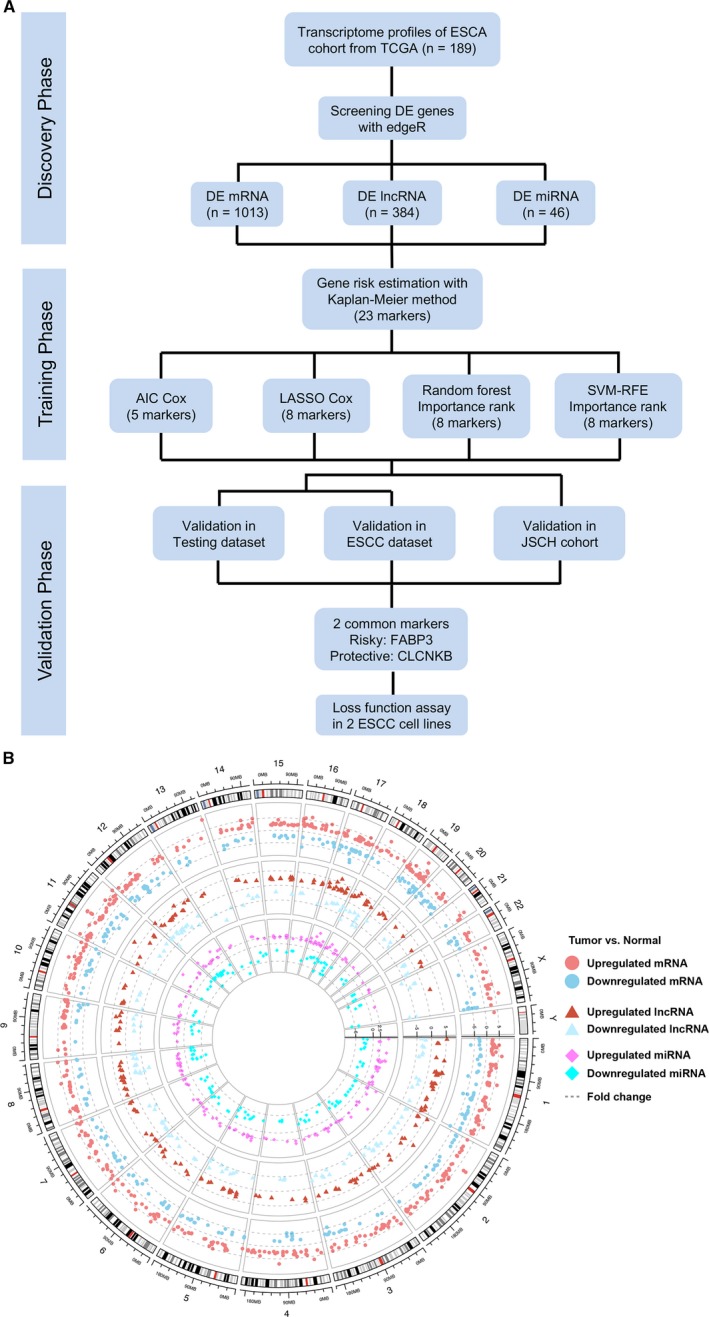
Selection of candidate prognostic markers for building signatures. A, Study flow chart. AIC, Akaike information criterion; DE, differentially expressed; ESCA, oesophageal cancer; ESCC, oesophageal squamous cell carcinoma; JSCH, JiangSu Cancer Hospital; LASSO, least absolute shrinkage and selection operator; SVM‐REF, Support Vector Machine‐Recursive Feature Elimination. B, Circos plot shows fold changes and genome locations for differentially expressed mRNAs, lncRNAs and miRNAs

The study flow chart is shown in Figure 1A, and we included three phases to identify and validate transcriptome signatures. In the discovery phase, TCGA ESCA project RNA and miRNA‐sequencing data were used to screen DEGs. In the training phase, two regularization semi‐parametric algorithms (AIC and LASSO Cox models) and two machine learning algorithms (RFS and SVM classifiers) were selected to conduct prognostic models and narrow markers. In the validation phase, four prognostic models were validated in testing and ESCC datasets, and loss‐of‐function assay identified oncogenic function of FABP3.

To construct the prognostic transcriptome signatures, we obtained DEGs from TCGA ESCA cohort including 187 ESCA specimens and 13 adjacent normal tissues. In total, under the threshold of FDR < 0.01 and |log2 (foldchange)| ≥ 2, 1013 mRNAs (Figure 1B, outer track), 384 lncRNAs (Figure 1B, middle track) and 46 miRNAs (Figure 1B, inner track) showed differential expression profiles between ESCA and adjacent normal tissues. In detail, 642 mRNAs, 217 lncRNAs and 18 miRNAs were up‐regulated, while 371 mRNAs, 167 lncRNAs and 28 miRNAs decreased expression in tumour tissues.

### 3.3 Construction of prognostic signatures

**Figure 2:**
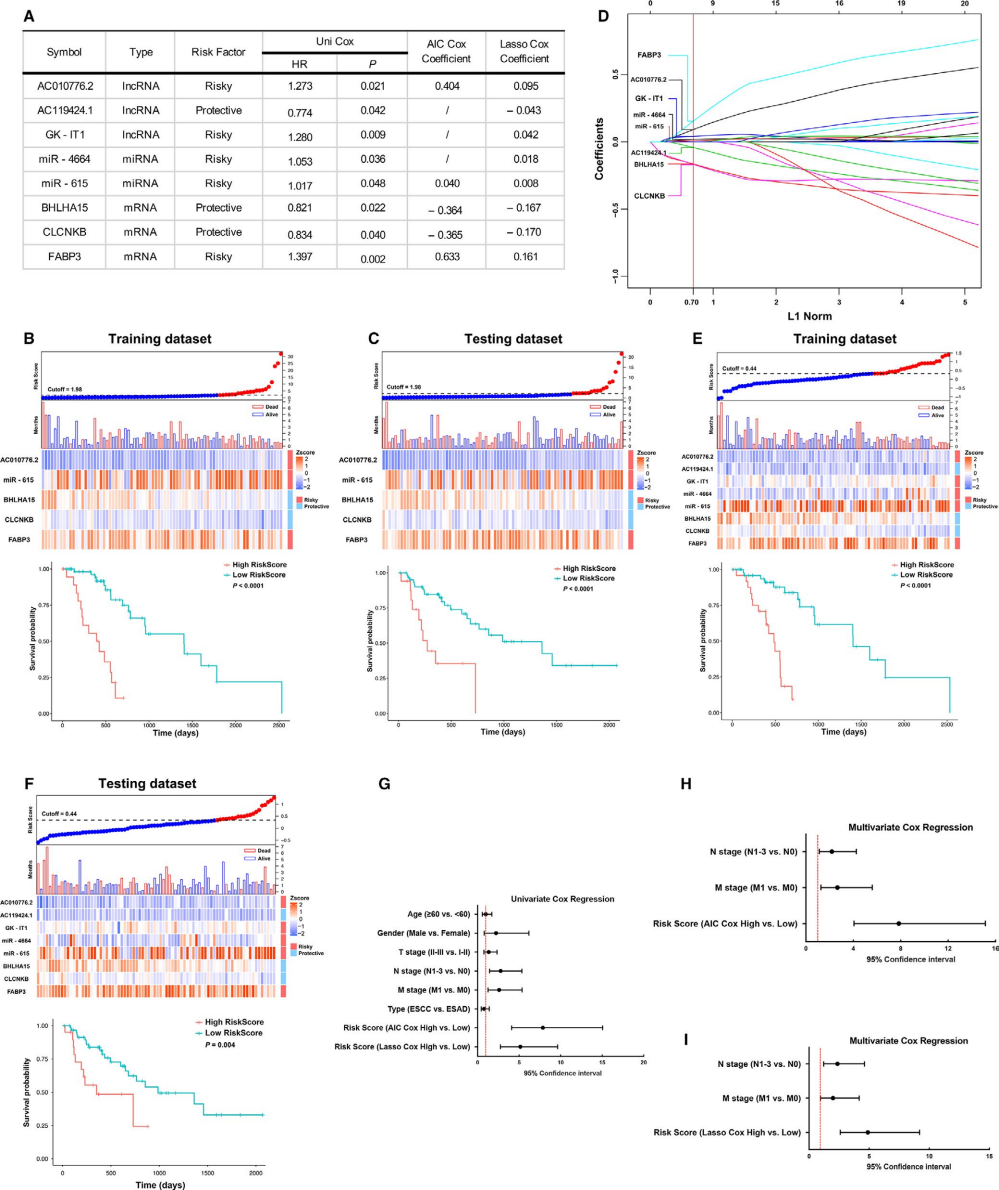
Construction and evaluation of prognostic signatures. A, The features of five common and three independent markers included in the AIC and LASSO Cox regression models. HR, hazard ratios; Uni, univariate. B, C, For the AIC Cox prognostic signature, waterfall plots and boxplots for distribution of risk scores and survival status, and heatmap plots for the expression level of each markers across samples. D, LASSO coefficient profiles of candidate markers. Each curve corresponds to a marker; the vertical line is drawn at the value log(*λ*) = −2.48 chosen by 10‐fold cross‐validation via minimum criteria. E, F, LASSO Cox prognostic signature in the training and testing datasets. G‐I, Univariate and multivariate Cox analyses of risk scores by two signatures mentioned above

We conducted the Kaplan‐Meier survival analyses to identify the association between the expression of DEGs and overall survival (OS). As a result, the significant DEGs were screened by AIC and LASSO Cox models to construct prognostic signatures (Figure 2A). In the AIC Cox model, the following formula was derived to calculate risk score for each patient: Risk score = (0.404 × expression of AC010776.2) + (0.040 × expression of miR − 615) − (0.364 × expression of BHLHA15) − (0.365 × expression of CLCNKB) + (0.633 × expression of FABP3). The X‐tile plots were used to generate an optimal selected cut‐off score (cut‐off = 1.98) to divide patients into high‐ and low‐risk score subgroups in the training dataset (Figure S1A). Then, we found patients with high risk score generally had worse OS than those with low risk (*P* < .0001) (Figure 2B), and the performance of AIC Cox prognostic signature was validated in the testing dataset by the same signature and cut‐off value (Figure 2C).

We constructed the other risk score formula with LASSO Cox model to verify the robustness of Cox regression in this datasets. 10‐fold cross‐validation via penalized maximum likelihood was used to compute regularization parameter lambda (Figure S1B). Eight (AC010776.2, AC119424.1, GK‐IT1, miR‐4664, miR‐615, BHLHA15, CLCNKB and FABP3) out of the 23 candidate markers were selected to construct a prognostic signature with optimal weighting coefficients (lambada: 0.084; Figure 2D). Compared with AIC Cox model, three additional markers (AC119424.1, GK‐IT1 and miR‐4664) were added to the LASSO risk score formula with optimal selected cut‐off value (cut‐off = 0.44) (Figure S1C). In the training dataset, patients with high risk score had worse OS than those with low risk (*P* < 0.0001) (Figure 2E), and then, the performance of LASSO Cox prognostic signature was also validated in the testing dataset (Figure 2F).

### 3.4 Predictive performance of two prognostic signatures

**Figure 3:**
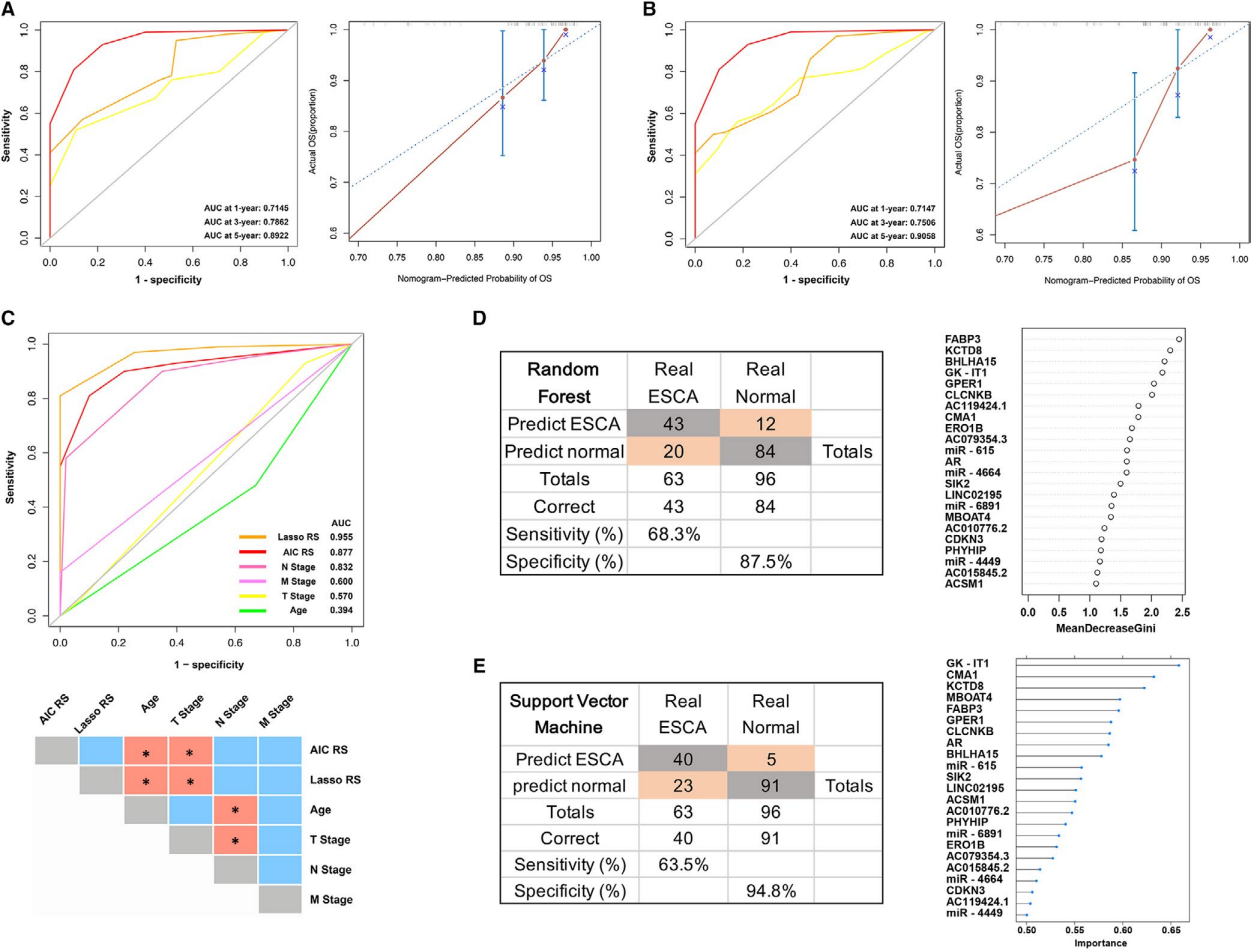
Predictive performances of prognostic signatures, Random Forest and Support Vector Machine algorithms. A, B, Time‐dependent receiver operating characteristic (ROC) curves in the whole ESCA cohort, and area under the curve (AUC) at 1‐, 3‐ and 5‐year curves were calculated according to the AIC and LASSO Cox regression signatures. The calibration plots for nomogram models represent ideal and observed nomograms, respectively. C, Comparisons of the prognostic accuracy among two signatures, age and TNM stages. AUCs were calculated and compared by the bootstrap method. D, Confusion tables of binary results of the Random Forest model in the whole cohort. Random Forest‐Feature Selection (RFS‐FS) is based on mean decreased Gini index. E, Confusion tables of binary results of the SVM model in the whole cohort. Recursive Feature Elimination (RFE) is used to estimate variable importance. *P < .05

To further validate the performance of these two prognostic signatures, univariate and multivariate Cox proportional hazard models were performed on risk scores and clinicopathological features (Figure 2G). Both the AIC and LASSO prognostic signatures were identified as independent prognostic factors (Figure 2H‐I). Accumulative effects of these two prognostic signatures were also assessed with time‐dependent ROC analysis. The results demonstrated that AUC for 1‐, 3‐ and 5‐year OS were 0.7145, 0.7862 and 0.8922 in AIC prognostic signature (Figure 3A, left), and 0.7147, 0.7506 and 0.9058 in LASSO prognostic signature (Figure 3B, left), respectively.

Additionally, we constructed nomogram predictive models, considering risk scores and clinicopathological features, to predict survival probability of ESCA patients. Nomograms were generated to predict 1‐, 3‐, and 5‐year OS in the whole ESCA cohort, including AIC Cox model (Figure S1D upper) and LASSO Cox model (Figure S1D lower). The calibration plots for the 5‐year OS rate were predicted well in both Cox models (concordance index: 0.808 in AIC Cox model, 0.760 in LASSO Cox model; Figure 3A,B, right).

Furthermore, we compared the predictive accuracy among prognostic factors (Figure 3C upper). The results demonstrated that no significant differences were detected among AUCs of N stage (AUC: 0.832), AIC risk scores (AUC: 0.877) and LASSO risk scores (AUC: 0.955) (Figure 3C, lower). However, AIC and LASSO models had significantly high AUCs than other single clinicopathological risk factors (*P* < .05), except for the lymph node and distant metastasis (*P* > .05) (Figure 3C, lower).

### 3.5 Prognostic prediction with Random Forest and Support Vector Machine algorithms

Machine learning algorithms are widely used in biomarkers  based prediction models at present. We applied the RFS‐FS and SVM‐RFE to detect the association between the expression of DEGs and overall survival. We firstly used RFS‐FS to narrow down markers in the training dataset via 200 growing trees (Figure S2A). Applying the model yielded a sensitivity of 75.0% and specificity of 83.0% for ESCA in the training dataset of 32 events and 47 censors (Figure S2C), and a sensitivity of 61.3% and specificity of 91.8% in the testing dataset of 31 events and 49 censors (Figure S2D). Then, the SVM‐RFE was also applied in the training dataset via cross‐validated accuracy (Figure S2B). This model indicated a sensitivity of 53.1% and specificity of 97.9% in the training dataset (Figure S2E), and a sensitivity of 74.2% and specificity of 91.8% in the testing dataset (Figure S2F).

These results demonstrated that the Random Forest model has a better performance compared with the SVM model in the training dataset, but worse in the testing dataset. Then, we measured variable importance in RFS model by Gini index which revealed that FABP3 is the most important marker (Figure 3D). Additionally, importance measures in SVM model via Recursive Feature Elimination (RFE) showed that GK‐IT1 ranks the top place (Figure 3E).

### 3.6 Predictive performance in the internal ESCC dataset and the Jiangsu Cancer Hospital ESCC cohort

**Figure 4:**
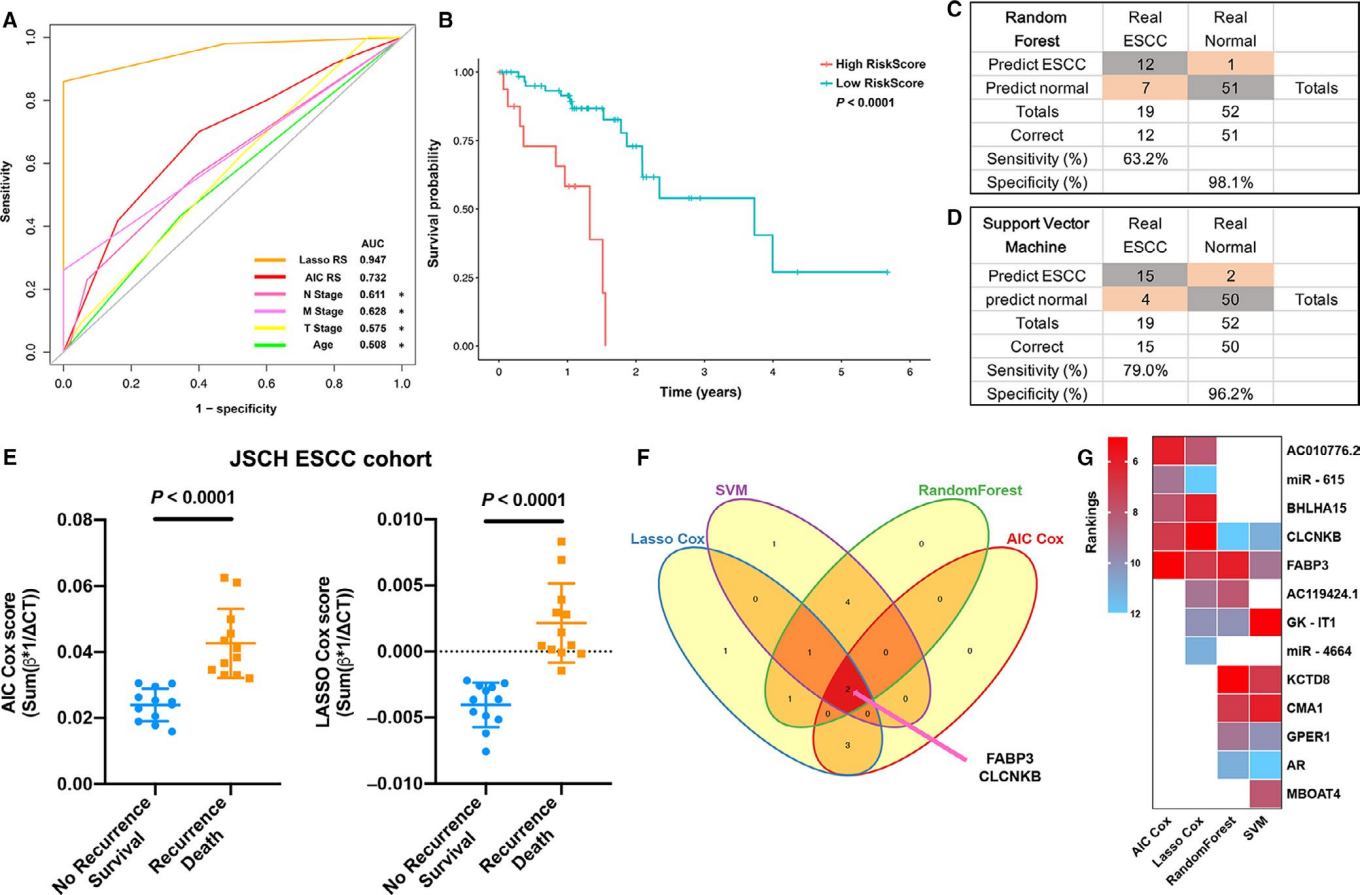
Predictive performance in the internal oesophageal squamous cell carcinoma (ESCC) dataset and the validation cohort, and the filter of novel markers. A, Time‐dependent ROC in the internal ESCC dataset, and AUCs were calculated and compared among predictive signatures, age and TNM stages by the bootstrap method. B, Kaplan‐Meier survival analysis based on the LASSO Cox model risk score in the ESCC patients. C, D, Confusion tables of binary results of the RFS and SVM models in the ESCC patients. E, Prognostic risk scores of two signatures in the JSCH (Jiangsu Cancer Hospital) cohort (2‐year prognosis). F, Venn plot shows the common marker FABP3 and CLCNKB. G, Heatmap of marker rankings. The Cox regression models rank the markers by coefficients, and RFS‐FS and SVM‐RFE were used by the machine learning algorithms. *P < .05

Oesophageal squamous cell carcinoma accounts for more than 90% ESCA patients in China,^8^, ^23^ so we compared the AUCs among predictive signatures and prognostic factors in the ESCC dataset with time‐dependent ROC analyses (Figure 4A). The results revealed that LASSO model (AUC: 0.947) has a higher AUC than AIC model (AUC: 0.732) and is significantly better than other single clinicopathological indicators (*P* < .05) (Figure 4A). The survival analysis showed that ESCC patients with high risk score had worse OS than those with low risk (*P* < 0.0001) in the LASSO model (Figure 4B). Furthermore, we detected the performance of two machine learning algorithms in the ESCC datasets. The RF model got a better specificity (98.1%, Figure 4C), and the SVM model showed a better sensitivity (79.0%, Figure 4D). The results suggested that machine learning models are stable predictors along with the LASSO model (specificity 98.0%, sensitivity 86.5%) in the ESCC patients.

To further validate the performance of the two prognostic signatures, we detected the expression of prognostic markers in 24 ESCC patients from Jiangsu Cancer Hospital cohort (Figure S3A). The prognostic signatures demonstrated that ESCC patients with poor prognosis got significant higher risk scores than survival patients (*P* < .0001) (Figure 4E).

### 3.7 FABP3 promotes malignant progression of oesophageal squamous cell carcinoma cells

**Figure 5:**
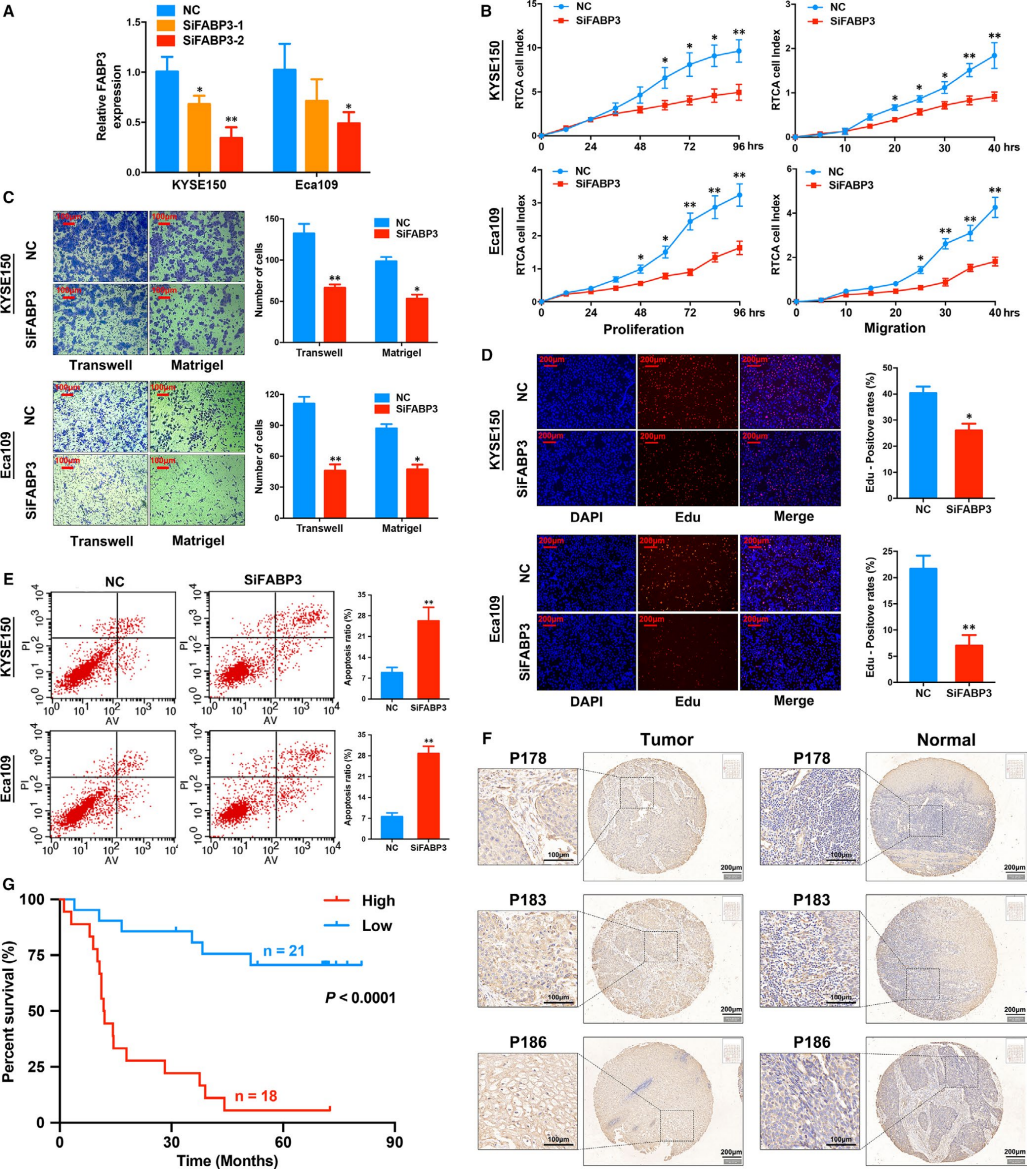
FABP3 promotes malignant progression of oesophageal squamous cell carcinoma cells. A, qRT‐PCR analyses of FABP3 RNA expression after treatment with two siRNAs. B, Cell proliferation and migration detected by Real Time xCELLigence Analysis system (RTCA) system. C, D, FABP3 promotes migration, invasion and proliferation of KYSE150 and Eca109 cell lines. E, FABP3 inhibits cell apoptosis. F, The expression of FABP3 was analysed by immunohistochemical (IHC) score on ESCC tissue. G, Kaplan‐Meier survival analysis based on immunohistochemical (IHC) score of ESCC patients in tissue microarray (TMA). *P < .05; **P < .01

In summary, after combining markers in the AIC Cox, LASSO Cox, RFS‐FE and SVM‐RFE models, 2 common markers were identified (risky marker: FABP3; protective marker: CLCNKB; Figure 4F). In addition, risk or importance of markers was compared in each model, and we found FABP3 achieved top few rankings in the all models (Figure 4G). To identify the properties of the novel marker FABP3, we designed two siRNAs to investigate the biological function of FABP3, and the expression of FABP3 was found significantly down‐regulated by siRNA‐2 in both ESCC cell lines (Figure 5A).

Next, we found the knockdown of FABP3 greatly suppresses the proliferation ability of KYSE150 and Eca109 cells by using RTCA proliferation and migration assays (Figure 5B). Additionally, the transwell and matrigel assays showed that silencing FABP3 significantly impairs the migration and invasion capabilities of KYSE150 and Eca109 cells (Figure 5C). Edu assays validated the results of the RTCA proliferation assay (Figure 5D), and the knockdown of FABP3 significantly promoted the apoptosis in the ESCC cell lines (Figure 5E). Collectively, our results suggested that FABP3 could promote the proliferation and migration abilities of ESCC cell lines. FABP3 expression was then detected by IHC using the TMA of 39 ESCC cases (Table S1). Overexpression of FABP3 in ESCC was validated by IHC scores in TMA (Figure 5F and Figure S3D). In addition, Kaplan‐Meier survival analysis showed that patients with higher levels of FABP3 (cut‐off: IHC score ≥ 8) had a shorter overall survival (*P* < .0001; Figure 5G).

## 4 DISCUSSION

Oesophageal cancer is a clinically challenging disease with a considerable decline in health‐related quality of life and a poor prognosis.^24^ Presently, traditional prognostic factors including tumour stage, tumour subsite and histology are hard to explain verified clinical outcome.^7^ In this study, we examined the transcriptome of ESCA and adjacent non‐tumour tissues from TCGA, and then constructed prognostic signatures which were closely related to the prognosis of ESCA patients. Our data showed that the LASSO signature developed in this study could stratify ESCA patients into good and poor survival groups effectively. These results were further validated in the internal ESCC dataset and external independent ESCC cohort. Furthermore, FABP3, the most heavily weighted marker in prognostic signature, was identified as a novel oncogenic gene in ESCC.

Using the signatures, high‐risk patients should be advised of the adjuvant treatment in addition to traditional surgery, especially in ESCC patients with the high LASSO signature risk score. However, the LASSO model reached a high level of predictive performance by a significant number of markers, which is inconvenient for the clinical application. FABP3 and CLCNKB were two common markers identified by all four algorithms, and the single‐factor risk of these two markers was validated in TCGA (Figure S3B,C). Based on the results of risk analyses and loss‐of‐function assay, detecting the expression level of FABP3 could be an option and alternative. Thus, the whole or part of the signature could help guide individualized adjuvant therapy schedules after traditional surgery.

Presently, high‐throughput sequencing data based prognostic signatures have been applied in many cancer types, such as colon cancer and hormone receptor positive (HR+) breast cancer.^17^, ^25^, ^26^ Cheong et al identify that a four‐mRNA signature is significantly associated with recurrence risk in multi‐centre gastric cancer cohorts.^27^ Although a few previous studies have demonstrated that mRNA 28, lncRNA 8 and miRNAs 29, 30 expression profiles are related to recurrence‐free survival (RFS) and OS in ESCC, whether combining all types of RNA markers could improve the performance of prognostic signatures still remains unknown. Additionally, few studies applied two or more algorithms to screen risky markers in ESCA, which is a significant bias for constructing signatures. In this study, we used two machine learning models to estimate the robustness of the Cox regression models.

We noted that FABP3 is the only risky marker identified by all four algorithms, AIC Cox regression, LASSO Cox regression, RFS‐FS and SVM‐RFE. The fatty acid–binding protein (FABP) family is involved in fatty acid signalling pathway, which is one of the most importantly involved pathways in cancer development.^31^ Tang et al showed that the high expression of FABP3 is correlated with poor prognosis in non–small‐cell lung cancer.^32^ Our results showed that FABP3 promotes the proliferation and migration of ESCC cell lines. The other common marker, protective marker CLCNKB is found predominantly expressed in the kidney and was demonstrated to be down‐regulated in clear cell renal cell carcinoma.^33^, ^34^ Compared with FABP3, the risk weighting of CLCNKB was less than FABP3 in the Cox regression models, and CLCNKB ranked lower than FABP3 in the both RFS‐FS and SVM‐RFE models. In addition, the protective function of CLCNKB is still need to be validated with function assays. Other included markers, such as miR‐615 35, 36 and BHLHA15,^37^ have been reported to be associated with the risk of gastric and some other cancers. Thus, the all new ESCA markers, discovered in the Cox regressions, RFS‐FS and SVM‐RFE, are worthy of further studies.

Our study had several limitations as well. First, the biologic mechanism of other prognostic markers, such as AC010776.2, GK‐IT1 and CLCNKB, was still unknown. Second, it could be better if the external independent validation dataset had a greater sample size. Importantly, prospective studies are required to further validate our findings.

In summary, we combined four algorithms, included two types of adjusted Cox regressions and two machine learning algorithms, to investigate RNA‐Seq, miRNA‐Seq and adjuvant clinical data of ESCA. Our results demonstrated that constructed signatures are potential prognostic tools to predict mortality risk in ESCA and ESCC, and FABP3 is a novel biomarker and newly identified oncogenic gene in ESCC.

## CONFLICT OF INTEREST

The authors have declared that no competing interest exists.

## AUTHORS’ CONTRIBUTIONS

ZQ, WSW and LTY contributed to the design of the study. WSW, LTY, FPQ and HCC performed the experiments. LTY, WSW, MZF, XWZ and XWJ contributed to the writing of the manuscript. LTY, YR, WSW, XYT and XL contributed to the material support of the study. All authors read and approved the final manuscript.

## Data Availability

The data that support the findings of this study are openly available in https://portal.gdc.cancer.gov/.
