# Supplementary figures and images for: Four transcription profile–based models identify novel prognostic signatures in oesophageal cancer
Source: J Cell Mol Med. 2019 Nov 19;24(1):711–21. doi: 10.1111/jcmm.14779 (PMC6933393; doi:10.1111/jcmm.14779)

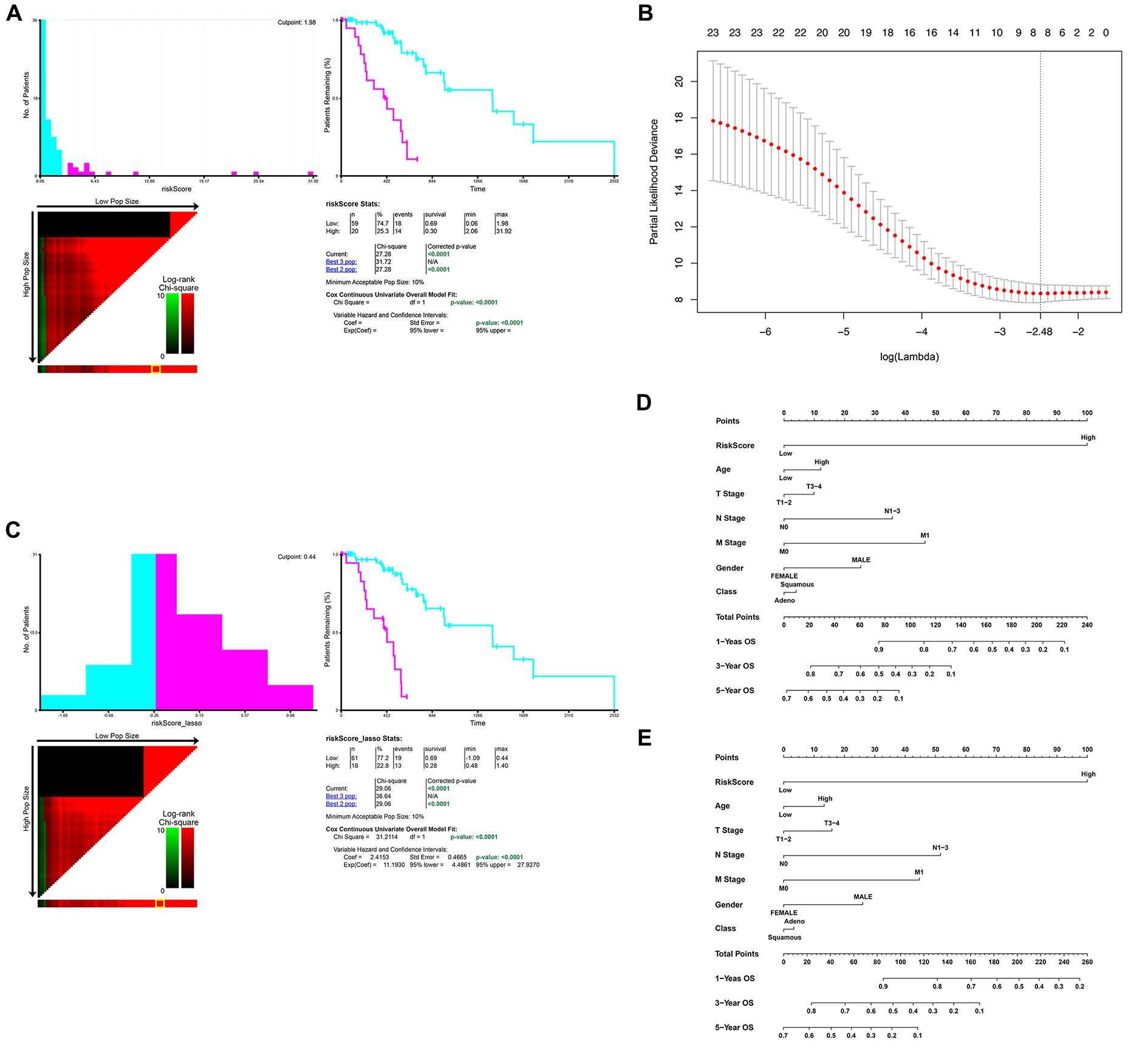

Supplement: Supplementary file 1 [file JCMM-24-711-s001.tif]

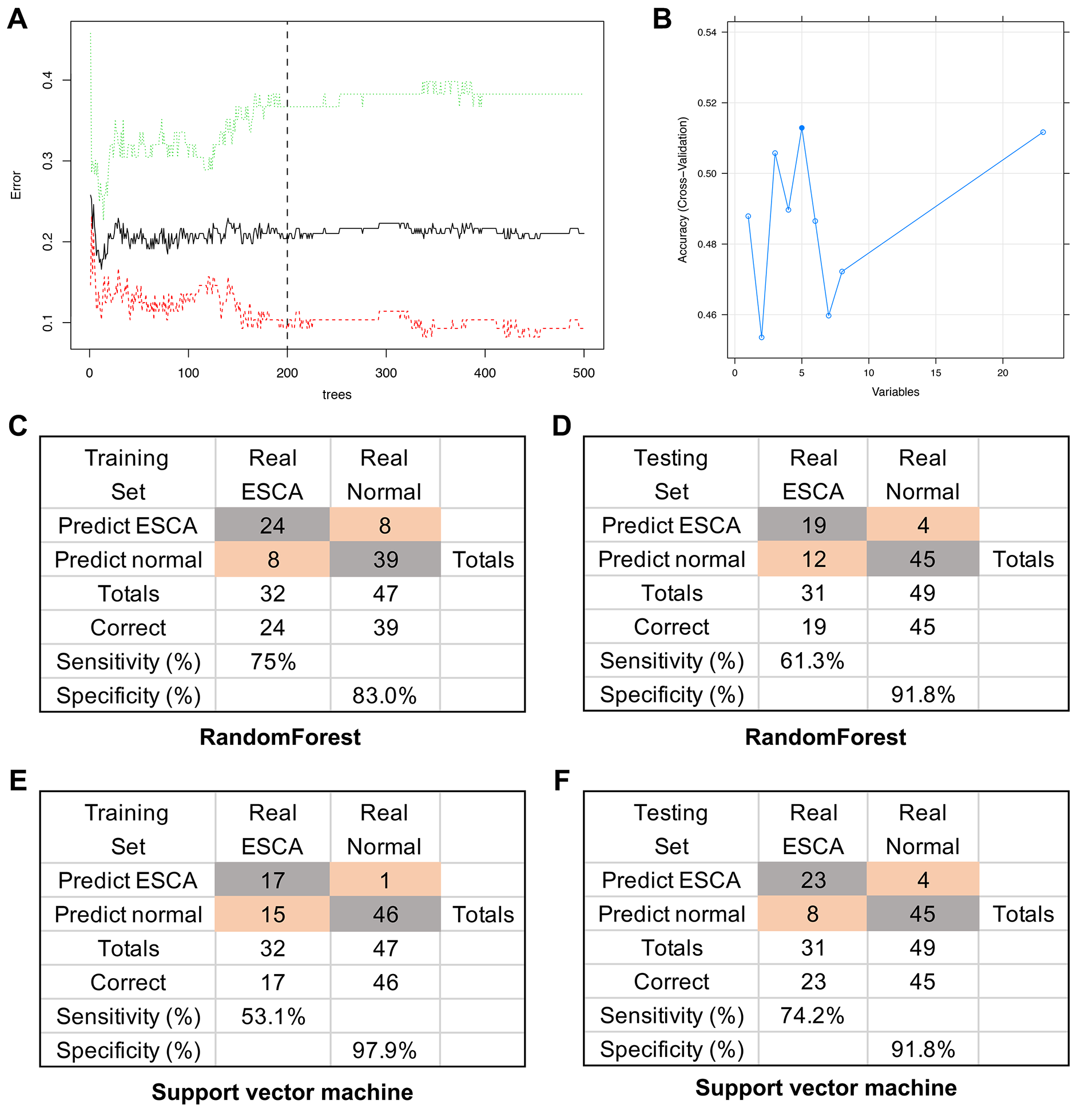

Supplement: Supplementary file 2 [file JCMM-24-711-s002.tif]

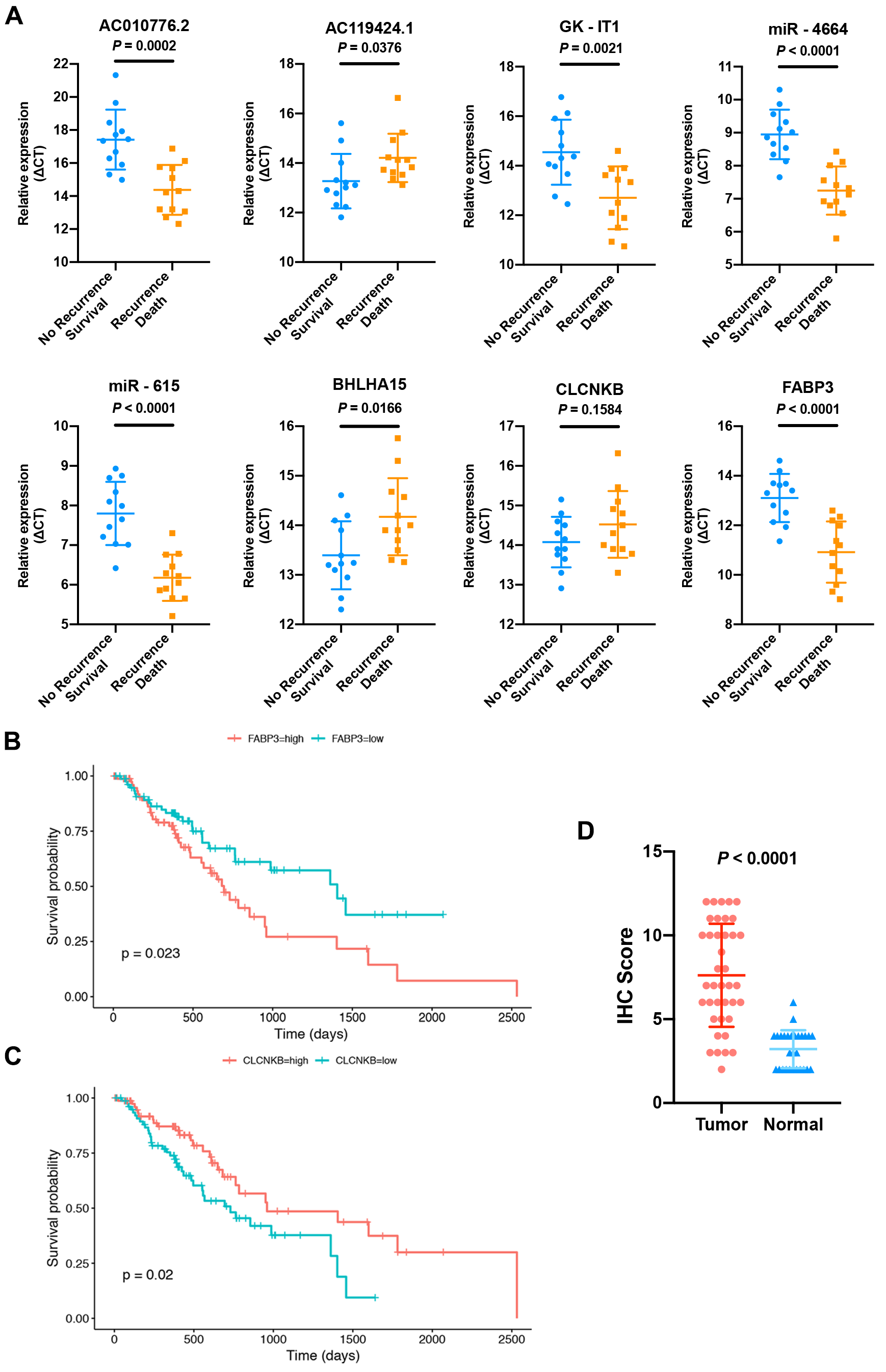

Supplement: Supplementary file 3 [file JCMM-24-711-s003.tif]
