# Supplementary material for: Four transcription profile–based models identify novel prognostic signatures in oesophageal cancer
Source: J Cell Mol Med. 2019 Nov 19;24(1):711–21. doi: 10.1111/jcmm.14779 (PMC6933393; doi:10.1111/jcmm.14779)
Supplement: Supplementary file 4 [file JCMM-24-711-s004.docx]

Supplementary table S1.

| Patient ID | Gender | Age | Histology | pTNM stage | Size | Lymph metastasis (1=yes，0=no) | Survival status (1=death，0=sensor） | Overall survival（Months） | IHC socre (Tumor) | IHC socre (Normal) |
| --- | --- | --- | --- | --- | --- | --- | --- | --- | --- | --- |
| 178 | Male | 52 | SCC | T3N1M0 | 3*2*2cm | 1 | 1 | 11.20 | 11.00 | 2.00 |
| 179 | Male | 48 | SCC | T2N1M0 | 5*2.5*2cm | 1 | 1 | 10.13 | 11.00 | - |
| 180 | Female | 45 | SCC | #N/A | 4.5*3*1.5cm | 1 | 1 | 3.07 | 10.00 | 4.00 |
| 181 | Male | 47 | SCC | T3N1M0 | 5*3*0.5cm | 1 | 1 | 51.27 | 6.00 | - |
| 182 | Male | 73 | SCC | T3N0M0 | 2*1*0.5cm | 0 | 1 | 35.50 | 4.00 | 2.00 |
| 183 | Male | 50 | SCC | T3N1M0 | 4*3*2cm | 1 | 1 | 37.57 | 12.00 | 4.00 |
| 184 | Male | 52 | SCC | T3N1M0 | 5*4*1cm | 1 | 0 | 77.20 | 5.00 | 4.00 |
| 185 | Male | 79 | SCC | T3N0M0 | 4*2*1cm | 0 | 1 | 16.60 | 7.00 | 4.00 |
| 186 | Male | 60 | SCC | T3N0M0 | 3.5*2.5*1.2cm | 0 | 1 | 28.23 | 10.00 | 4.00 |
| 187 | Male | 42 | SCC | T3N0M0 | 5*4*1cm | 0 | 0 | 71.63 | 7.00 | 2.00 |
| 188 | Male | 73 | SCC | T3N0M0 | 6*4*2cm | 0 | 0 | 72.57 | 8.00 | 4.00 |
| 189 | Female | 62 | SCC | T2N1M0 | 3.5*1.5*1cm | 0 | 1 | 38.33 | 6.00 | 4.00 |
| 190 | Male | 75 | SCC | #N/A | 3*2*1.5cm | 1 | 1 | 1.10 | 9.00 | 6.00 |
| 191 | Male | 36 | SCC | T3N1M1 | 5*4*1cm | 1 | 1 | 3.93 | 7.00 | 4.00 |
| 192 | Male | 38 | SCC | T2N0M0 | 5*3*1cm | 0 | 0 | 71.43 | 6.00 | 4.00 |
| 193 | Male | 59 | SCC | T3N1M0 | 2*1.2*1cm | 1 | 1 | 44.20 | 10.00 | 3.00 |
| 194 | Male | 68 | SCC | T3N1M0 | 6*3.5*1.5cm | 1 | 1 | 14.27 | 12.00 | - |
| 195 | Male | 58 | SCC | T3N1M0 | 4*3*1.2cm | 1 | 0 | 72.27 | 3.00 | 2.00 |
| 196 | Female | 59 | SCC | #N/A | 2*1.5*1.2cm | 0 | 1 | 14.40 | 10.00 | 5.00 |
| 197 | Male | 70 | SCC | T3N1M0 | 6*4*2cm | 0 | 1 | 39.07 | 10.00 | 2.00 |
| 198 | Male | 69 | SCC | T3N1M0 | 3*4*0.8cm | 1 | 1 | 10.67 | 11.00 | - |
| 199 | Male | 79 | SCC | T3N1M0 | 4*3*1.5cm | 1 | 1 | 12.00 | 12.00 | - |
| 200 | Male | 64 | SCC | T3N1M0 | 2*1.8*1.5cm | 0 | 0 | 74.23 | 6.00 | 3.00 |
| 201 | Male | 55 | SCC | T3N1M0 | 5*5*1.8cm | 1 | 0 | 81.07 | 5.00 | 2.00 |
| 202 | Female | 60 | SCC | #N/A | 3*1*0.5cm | 0 | 1 | 11.77 | 11.00 | 2.00 |
| 203 | Female | 54 | SCC | #N/A | 4*4cm | 0 | 0 | 71.77 | 3.00 | 2.00 |
| 204 | Male | 55 | SCC | T2N0M0 | 4*3*1cm | 0 | 0 | 71.77 | 4.00 | 3.00 |
| 205 | Male | 71 | SCC | T2N0M0 | 1.5*1*1cm | 0 | 1 | 10.57 | 7.00 | 4.00 |
| 206 | Male | 63 | SCC | T4N1M0 | 5*3*1.5cm | 0 | 1 | 17.93 | 10.00 | - |
| 207 | Female | 60 | SCC | #N/A | 5*3.5*0.5cm | 1 | 1 | 8.00 | 12.00 | - |
| 208 | Male | 60 | SCC | T3N1M0 | 3*3*2cm | 1 | 1 | 8.97 | 8.00 | - |
| 209 | Male | 43 | SCC | T3N1M1 | 5*3*2.5cm | 1 | 0 | 70.60 | 7.00 | - |
| 210 | Male | 61 | SCC | T3N1M0 | 4*2*1.6cm | 1 | 0 | 53.10 | 6.00 | 4.00 |
| 211 | Male | 73 | SCC | #N/A | 7*3*0.5cm | 1 | 0 | 71.03 | 5.00 | - |
| 212 | Male | 54 | SCC | T2N1M0 | 3*1.5*1.5cm | 1 | 1 | 11.17 | 12.00 | - |
| 213 | Male | 50 | SCC | #N/A | 4.5*2*1cm | 1 | 0 | 31.20 | 6.00 | 4.00 |
| 214 | Male | 66 | SCC | T3N0M0 | 6*4*2cm | 0 | 0 | 71.30 | 3.00 | 2.00 |
| 215 | Male | 66 | SCC | T3N0M0 | 4*2*1cm | 0 | 0 | 71.30 | 3.00 | 2.00 |
| 216 | Male | 62 | SCC | T3N0M0 | 3.5*3*2cm | 0 | 0 | 71.30 | 2.00 | 2.00 |

Supplementary table S1. Clinical features and immunohistochemistry (IHC) scores of FABP3 for ESCC patients in the tissue microarray (TMA). SCC, Squamous Cell Carcinoma.
